# P-974. An Embedded Transplant Infectious Diseases Fellowship Track Is Appealing to Clinical and Research-Focused Fellows

**DOI:** 10.1093/ofid/ofae631.1164

**Published:** 2025-01-29

**Authors:** Monica Fung, Brian S Schwartz, Jennifer M Babik

**Affiliations:** UCSF, San Fransisco, California; University of California, San Francisco, San Francisco, CA; UCSF, San Fransisco, California

## Abstract

**Background:**

Transplant Infectious Diseases (TID) training is commonly completed as a separate fellowship following ACGME ID fellowship. There is limited evidence on the impact of an embedded TID training track within ID fellowship.

Fellow Interest and Preferences for Transplant Infectious Disease Training by Career Focus

**Figure:**
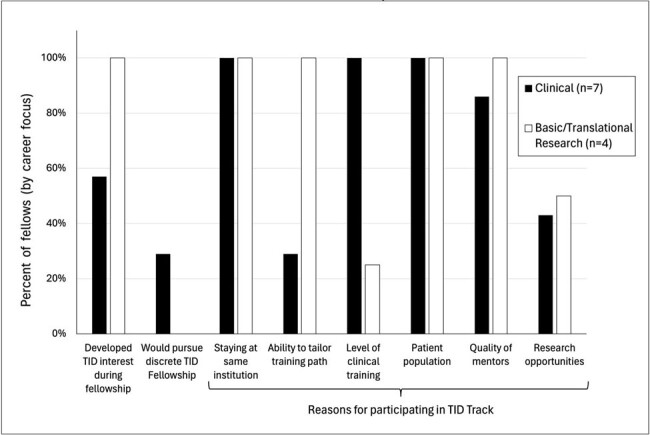

**Methods:**

Our TID track, created in 2016, aligns with existing standards for TID training. Fellows may complete the track within a 2-year ACGME fellowship or as part of a longer research track. Current and prior fellows of our TID track were anonymously surveyed about TID track interest/impact and career goals.

**Results:**

All 11 prior and current track fellows completed the survey. 64% of fellows (n=7) have or plan to pursue careers in clinical medicine, with a secondary focus on medical education (73%, n=8) and/or research (73%, n=8). 36% (n=4) have or plan to pursue primary careers in basic/translational research with a secondary focus in clinical medicine (Figure 1). Only 3 (26%) fellows were committed to TID prior to fellowship, with most (73%) developing an interest in TID during fellowship. Trainees cited interesting clinical cases (100%), mentorship/role models (73%), and research opportunities (55%) as reasons for their interest in TID training.

If an embedded TID fellowship were not offered, 18% (n=2, both with primary clinical focus) of fellows would have pursued a discrete TID year. All fellows cited the ability to stay at the same institution and patient population as factors for participating in our track, with 91% selecting quality of mentors and 73% level of clinical training. 55% of all fellows, and 100% of fellows pursuing careers in basic/translational science, specified the ability to tailor training path as a reason for participating in our track.

Since 2022, we have had 41% (7/17) of the fellows in our ID fellowship participate in the TID track. All track graduates (n=6) are currently in academic positions specializing in TID.

**Conclusion:**

Among ID training programs with adequate patient population and clinical expertise/mentors to provide specialized training in TID, an embedded TID Track within ID fellowship may increase recruitment into TID, particularly among fellows pursuing a career in basic/translational research and not initially interested in TID prior to fellowship. Results are limited by our single-center experience.

**Disclosures:**

**All Authors**: No reported disclosures

